# Fatty acid metabolic reprogramming in the tumor microenvironment: Unraveling mechanisms and therapeutic prospects

**DOI:** 10.1016/j.gendis.2025.101772

**Published:** 2025-07-14

**Authors:** Wenxin Zhang, Peiwen Wang, Guiqiang Yuan, Fusheng Liu, Guishan Jin, Junwen Zhang

**Affiliations:** a Brain Tumor Research Center, Beijing Neurosurgical Institute, Capital Medical University, Beijing 100070, China; b Department of Neurosurgery, Beijing Tiantan Hospital Affiliated to Capital Medical University, Beijing 100070, China

**Keywords:** Cancer progression, Cell interaction, Fatty acid, Immunotherapy, Tumor microenvironment

## Abstract

Lipid metabolic reprogramming has emerged as a hallmark in cancer research, especially that of fatty acids (FAs). It promotes the effective utilization of the limited nutrients in the tumor microenvironment (TME) by the cells and has considerably been associated with immune escape. Tumor cells exhibit enhanced FA uptake, synthesis, and oxidation for metabolic adaptation, and non-tumor cells also undergo FA metabolic remolding in the TME. Owing to the essential role of FA metabolism in TME, the associated critical enzymes may be targeted for developing novel therapeutic approaches. This review aims to comprehensively summarize the FA metabolic landscapes in various cancers and FA-related molecular changes, FA metabolic reprogramming in different cells in the TME to identify potential targets, and FA-related cell interactions and underlying mechanisms in the TME. The findings of this study may provide insights into exploring the intricate FA metabolism–TME adaptation interplay to uncover potential metabolic targets of therapeutic significance for combinatorial strategies and enhancing immunotherapy.

## Introduction

Fatty acids (FAs), carboxylic acids of various saturated and unsaturated long-chain hydrocarbons, are an integral component of various lipids, including triglycerides, phospholipids, and glycolipids, and regulate physiological or pathological functions in different cells by serving as an energy source and cellular-signaling mediator.^1^ During the pathogenesis of cancer, cancer cells have been reported to exhibit enhanced lipid uptake, generation, storage, and utilization to adapt to the changing microenvironment, which is known as lipid reprogramming.^2^ Because FAs provide necessary energy sources under metabolic stress conditions, cancer cells exhibit an increased dependence on FA *de novo* biosynthesis and exogenous uptake to maintain their rapid proliferation rate.^3^ In the tumor microenvironment (TME), the tumor cells with the highest proportion modify the composition of other cells, making them more conducive to tumor survival. Overall, FA metabolic reprogramming in cancers can induce intricate molecular changes and cell interactions through cytokine secretion, thereby leading to changes in the TME. This review comprehensively discusses FA-associated metabolic changes in cancers and cell interactions among tumor and non-tumor cells in the TME. Additionally, the review focuses on identifying new targets for combination therapy with metabolic reprogramming and immunotherapy.

## Fatty acid metabolism in cancers

### Fatty acid metabolism-related enzymes

**Figure 1:**
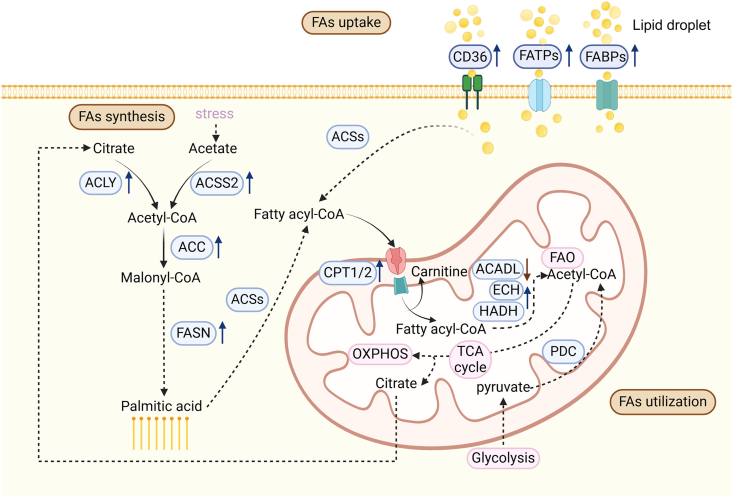
Schematic diagram of FA metabolic changes in cancer. Elevated levels of CD36, FATPs, and FABPs increase FA uptake. Meanwhile, intracellular FAs originate from citrate or acetate under different circumstances and are finally composed of palmitic acid, catalyzed by enzymes such as ACLY, ACSS2, ACC and FASN. Both exogenous and endogenous FAs become sources of fatty acyl-CoA, which is then transferred into mitochondria via CPT to participate in FAO for energy production. FA, fatty acid; CoA, coenzyme A; CD, cluster of differentiation; FATP, FA transport protein; FABP, FA-binding protein; ACLY, adenosine triphosphate–citrate lyase; ACSS2, acetate by acetyl-CoA synthetase 2; ACC, acetyl-CoA carboxylase; FASN, FA synthase; CPT, carnitine acyl transferase; ACADL, long-chain acyl-CoA dehydrogenase; ECH, enoyl-CoA hydratase; HADH, L-3-hydroxyacyl-CoA dehydrogenase; FAO, FA oxidation; TCA, tricarboxylic cycle; PDC, pyruvate dehydrogenase complex; OXPHOS, oxidative phosphorylation; ACSs, acyl-CoA synthetase. All the illustrations in this article were created with Biorender.com.

The fatty acid (FA) metabolic process mainly includes the uptake, synthesis, and oxidation of FAs (Fig. 1). Mammalian cells typically obtain FAs through a direct exogenous uptake from the outside microenvironment or endogenous synthesis from acetyl-coenzyme A (CoA), which is obtained from the breakdown of glucose and glutamine.^4^

Furthermore, the cellular uptake of long-chain FAs (LCFAs) is facilitated by various proteins, which have been reported to be upregulated in cancers, namely FA translocase (FAT), also called the scavenger receptor cluster of differentiation 36 (CD36), plasma membrane FA-binding proteins (FABPs), and the six FA transport proteins (FATPs).^4^^,^^5^

Endogenous FAs are synthesized in the cytoplasm utilizing cytoplasmic acetyl-CoA obtained from either citrate by adenosine triphosphate (ATP)–citrate lyase (ACLY) or from acetate by acetyl-CoA synthetase 2 (ACSS2).^6^ Notably, after glycolysis-derived pyruvate enters the mitochondria, it is converted into acetyl-CoA by the pyruvate dehydrogenase complex (PDC) and then to citrate, which then enters the Krebs cycle. After entering the cytoplasm through the citrate–pyruvate cycle, citrate is then transformed into acetyl-CoA by ACLY.^7^ In contrast, under metabolic stress conditions such as lipid depletion or hypoxia, cells upregulate cytosolic ACSS2 to generate acetyl-CoA from acetate.^8^ Next, acetyl-CoA carboxylase (ACC), a key enzyme in palmitic acid (PA) synthesis, converts acetyl-CoA to malonyl-CoA, seven molecules of which then combine with one molecule of acetyl-CoA to form a molecule of PA. This reaction is catalyzed by the FA synthase (FASN) complex via seven cycles of the basic reactions of condensation, reduction, dehydration, and reduction. Up-regulated FA synthesis, particularly *de novo* synthesis, is characteristic of tumor cells,^9^^,^^10^ and increased FASN levels have been observed in breast, prostate, colon, ovarian, and endometrial cancers.^11^, ^12^, ^13^, ^14^, ^15^, ^16^

Reportedly, acyl-CoA synthetase initiates FA decomposition to form fatty acyl-CoA, which is transported into mitochondria by carnitine acyl transferase1 (CPT1). In the mitochondria, β-oxidation, the core step of FA decomposition, generates numerous ATP and acetyl-CoA molecules, which then enter the Krebs cycle or synthesize ketone bodies. FA β-oxidase in the mitochondrial matrix catalyzes the oxidation of fatty acyl-CoA through repeated four reactions via long-chain acyl-CoA dehydrogenase (ACADL), enoyl-CoA hydratase (ECH), L-3-hydroxyacyl-CoA dehydrogenase (HADH), and β-keto thiolase. Increased ATP generation due to upregulated FA oxidation (FAO) is a distinctive characteristic of cancer cells,^17^ and upregulated expression of FAO-related proteins has been reported in different human cancers.^18^, ^19^, ^20^, ^21^, ^22^, ^23^

Notably, FA anabolism and catabolism can co-occur at different parts of the tumor. For instance, tumor cells may synthesize new lipids to invade other central nervous system tissues when near nutrient-rich conditions, whereas those near hypoxic, nutrient-poor regions may consume lipids to produce ATP for survival.^24^

Increased FAT, FABP, and FATP expressions in cancer lead to the capture of more FAs, thereby increasing the lipid content to satisfy energy consumption for proliferation. The Cancer Genome Atlas database indicates that the CD36 gene is amplified or mutated in many tumors, including esophagogastric cancer, melanoma, and non-small cell lung cancer,^25^ and serves as an independent prognostic marker owing to its overexpression in the brain,^26^ breast,^27^ colon,^28^ gastric,^29^ and ovarian^30^ tumors.^31^ Pascual et al. reported significantly increased lipid uptake by CD36, along with CD36-induced FAO activation in oral squamous cell carcinoma (OSCC) metastasis,^32^ elucidating the pro-metastasis role of CD36-dependent lipid absorption in tumor progression.^31^ The twelve FABP family members are water-soluble small LCFA-binding proteins^33^ and are distributed across various tissues, such as hepatocytes, adipocytes, epidermis, and the brain.^34^ Reportedly, FABP3 (heart-type FABP), FABP4 (adipocyte FABP [A-FABP]), FABP5 (epidermal FABP [E-FABP]), and FABP7 (brain FABP) are upregulated in various tumors; the expression of FABP1 (liver FABP) has been shown to vary across tumors. Similarly, the FATP family has six members distributed across various tissues, with FATP1–4 showing pro-tumoral effects and FATP5 exhibiting tumor-inhibiting effects.^35^

The dysregulation of ACLY, ACSS2, and PDC has been shown to promote cancer progression via increased acetyl-CoA production.^36^ For instance, amplified or somatic-mutated ACLY has been found in various malignancies. The second isoform of ACSS, namely ACSS2, exhibits high alteration frequency or expression in different cancers, including uterine endometrioid carcinoma, esophagogastric cancer, breast cancer, glioblastoma multiforme, and liver cancer. High ACLY and ACSS2 expression are both correlated to poor prognosis in patients with cancer. Furthermore, phosphoinositide-dependent kinase (PDK)1, one of the four isoforms of PDK, is up-regulated in multiple cancer types and reprograms metabolism in cancer cells.

In eukaryotic cells, two ACC isoforms exist, namely, ACC1, which synthesizes malonyl-CoA for FA synthesis, and ACC2, which catalyzes malonyl-CoA-mediated inhibition of CPT1 and FA degradation. Owing to the Warburg effect, tumor cells tend to enhance lactate accumulation,^37^ which is widely correlated with tumor clinical stages and negative outcomes regarding patient survival.^38^ ACC1 has been studied in various cancer types and is considered an inhibition target in breast, liver, lung, and colon cancers.^39^ Additionally, high FASN expression has been detected in the tissues of patients with breast, lung, colorectal, prostate, and bladder cancers and in the sera of patients with pancreatic, colorectal, and breast cancer.^40^ Owing to the critical role of FASN in FA synthesis in most cancer cells, it is a well-studied cancer target, and 11 FASN inhibitors are being used at present.^41^

The long-chain acyl-CoA synthetase (ACSL) family comprises of five isoenzymes, namely, ACSL1 and ACSL3–6.^42^ ACSL3 facilitates tumor progression, whereas ACSL5 is involved in the pro-apoptotic sensing process of the cell and serves as a tumor suppressor.^43^^,^^44^ ACSL1 and ACSL4 are differentially expressed in different cancers, with ACSL4 being positively correlated with immune cell infiltration in various TMEs.^45^^,^^46^ The CPT1 enzyme family includes the canonical members CPT1A and CPT1B, which regulate the limiting step in FAO, along with CPT1C, whose catalytic activity remains elusive.^47^ CPT1A overexpression has been commonly associated with malignant progression in various tumors such as gastric, breast, prostate, ovarian, and lung cancers.^48^, ^49^, ^50^ Furthermore, bioinformatics analysis revealed that CPT1B is related to colorectal cancer (CRC) and muscle-invasive bladder cancer.^51^ Interestingly, CPT1C expression has been found to be nearly exclusive to neurons and stem cells,^52^^,^^53^ and is supposed to promote cancer cell survival and tumor growth,^54^ along with acting as a nutrient sensor in neurons to regulate metabolism and function.^47^ Higher CPT1C expression has been reported in non-small cell lung carcinoma, papillary thyroid carcinoma, and hepatocellular carcinoma (HCC) and has been associated with poor prognosis in patients with HCC and gastric, breast, ovarian, and lung cancers.^47^ After entering the mitochondria, acyl-CoA repeatedly undergoes a four-step process catalyzed by ACADL, ECH, HADH, and β-ketothiolase to complete β-oxidation. Reportedly, ACADL acts as a tumor suppressor in HCC^55^ and lung adenocarcinoma (LUAD),^56^ and it exhibits a low expression in these cancers. Additionally, ECH may play a dual role in cancer development, as it acts as a tumor promoter in different cancer types, including pancreatic, nasopharyngeal, colorectal, lung, prostate, breast, and gastric cancers, and as a tumor suppressor gene in others, including renal cell carcinoma, conjunctival melanoma, bladder cancer, and endometrial cancer.^57^^,^^58^ Reportedly, ECH1 is an important factor in the lymphatic metastasis of tumors.^59^ HADH expression has been found to be up-regulated in renal cell carcinoma, liver cancer, and gastric cancer, whereas it is downregulated in colon cancer.^60^

### Regulators of FA metabolic enzymes

Several molecules can indirectly regulate FA metabolism in cancers via the transcriptional control of the above FA metabolic-related enzymes.

As a member of the peroxisome proliferator-activated receptor family, PPARγ, which up-regulates the expression of FABP4, FASN, and ACLY, can promote FA uptake and FA synthesis.^61^^,^^62^ The activation of PPARγ has been proven to be an effective anti-tumor strategy in melanoma, pancreatic, liver, and lung cancer.^63^ Sterol regulatory element-binding proteins (SREBPs) induce the transcription of FA *de novo* synthesis-related genes, such as FASN, ACLY and ACC. In liver cancer, high expression of SREBP-1 is a predictor for tumor progression and patient survival.^64^^,^^65^ Carbohydrate response element binding protein (ChREBP), which promotes the transcription of FASN, ACC1, Stearoyl-CoA Desaturase 1(SCD1), and elongation-of-very-long-chain-fatty acids (ELOVL6), also showed an increased expression level in colon cancer tissue compared to healthy colon tissue.^66^ ChREBP contributes to colon cancer cell proliferation and cancer progression.^66^ Liver X receptors α and β (LXRα and LXRβ), which are nuclear receptors, indirectly regulate FA synthesis via the transcriptional control of SREBP1c and ChREBP.^65^^,^^67^ LXR agonists inhibited the cell proliferation and progression of lung, breast, colorectal, pancreatic and liver cancer.^68^

Other two members of PPAR family, PPARα and PPARβ/δ, can promote FAO^62^^,^^69^ process. Like PPARγ, PPARα is associated with tumor suppression in hepatocellular carcinoma and colorectal cancer.^70^ However, PPARβ/δ plays a dual role in different cancers, such as exerting a tumor-promoting effect on colorectal cancer.^70^ Peroxisome proliferator-activated receptor gamma coactivator-1α (PGC-1α), which interacts with PPARα to activate CPT1A and ACADL for elevated FAO, also has opposing roles in different subtypes of the same tumor or different cancer types.^71^

According to above FA metabolism changes in cancer, a summary of the various mechanisms in developing current FA metabolism-related drugs is displayed in Table 1. We discuss the mechanisms, targets and example drugs to provide information for future drug development and application.

**Table 1:** Mechanisms in developing FA metabolism-related drugs.

Mechanism	Target	Example Drugs
**1. Inhibition of FA uptake**		
CD36 blockade	CD36	SSO, FA6.152, JC63.1, Cyclic psap, VT1021^25^
FABPs inhibition	FABP4/5	SBFI-102/103/26, BMS309403^72^, ^73^, ^74^, ^75^
FATPs inhibition	FATP2/5	Lipofermata, Perphenazine^35^^,^^76^
**2. Inhibition of FA synthesis**		
ACLY inhibition	ACLY	SB-204990, Bempedoic acid, BMS-303141^77^, ^78^, ^79^
ACSS2 inhibition	ACSS2	VY-3-135, AD-8007 5584, MTB-9655^80^, ^81^, ^82^
ACC inhibition	ACC1/2	TOFA, PF-05175157^83^^,^^84^
FASN inhibition	FASN	TVB-2640/3166/3664, Orlistat, Cerulenin, C75^85^, ^86^, ^87^, ^88^, ^89^
**3. Inhibition of FA oxidation (FAO)**		
CPT1s inhibition	CPT1A/B	Etomoxir, Perhexiline^90^^,^^91^
ACSLs inhibition	ACSL1/3/4	Triacsin C, PRGL493^92^^,^^93^
**4. Inhibition of FA metabolic enzyme regulators**		
PPARs activation	PPARα/γ	Rosiglitazone, Pioglitazone, Fenofibrate^94^
SREBPs inhibition	SREBP1/2	Fatostatin, Betulin^95^
ChREBP inhibition	ChREBP	SBI-993^96^
LXRs activation	LXRα/β	LXR-623, GW3965^97^
PGC-1α inhibition	PGC-1α	SR-18292^96^

CD, Cluster of Differentiation; FABP, Fatty Acid-Binding Protein; FATP, Fatty Acid Transport Protein; ACLY, ATP-Citrate Lyase; ACSS2, Acyl-CoA Synthetase Short-Chain Family Member 2; ACC, Acetyl-CoA Carboxylase; FASN, Fatty Acid Synthase; CPT1, Carnitine Palmitoyltransferase 1; ACSL, Acyl-CoA Synthetase Long-Chain; PPAR, Peroxisome Proliferator-Activated Receptor; SREBP, Sterol Regulatory Element-Binding Protein; ChREBP, Carbohydrate-Responsive Element-Binding Protein; LXR, Liver X Receptor; PGC-1α, Peroxisome Proliferator-Activated Receptor Gamma Coactivator 1-Alpha.

## FA metabolic reprogramming in the TME

The TME is a dynamic and intricate niche harboring both tumor cells and dysfunctional non-cancerous cells embedded in an abnormal extracellular matrix (ECM). It encompasses a multitude of cell types, such as diverse immune cells, cancer-associated fibroblasts (CAFs), endothelial cells (ECs), pericytes, and various additional tissue-resident cell types.^98^ Immune cells in the TME mainly include tumor-infiltrating T cells (TILs), dendritic cells (DCs), tumor-associated macrophages (TAMs), myeloid-derived suppressor cells (MDSCs), and natural killer (NK) cells.^99^ The non-cellular portion of the TME includes ECM components, such as hyaluronan, collagen, fibronectin, and laminin; soluble proteins or peptides, such as enzymes, growth factors, chemokines, and other cytokines; and extracellular vesicles and metabolism products including FAs, cholesterols, and lactate. Taken together, these findings facilitate tumor development and progression. At the molecular level, both cellular and non-cellular components in the TME are of critical importance and are involved in intricate cell–cell interactions via paracrine and autocrine signaling involved in tumor progression.^100^ Consequently, various studies have investigated the correlation between important FA metabolic enzymes and TME changes, especially regarding immune infiltration, via a bioinformatics approach (Table 2).

**Table 2:** Bioinformatics analysis of key targets in fatty acid (FA) metabolism and tumor microenvironment (TME) changes.

Target	Tumor	TME change		Year
		Positive correlation	Negative correlation	
ACLY	HCC	CD4^+^T cell, monocyte, mDC, Treg, and neutrophil	CD8^+^ T cell and NK cell	2021^101^
ACSS2	CESC	CD4^+^T cell, macrophage, and B cell	CD8^+^ T cell, DC, and neutrophil	2021^102^
ACACA	HCC	T Helper cell, Tcm, Th2 cell, and eosinophil	CD8^+^ T cell, NK cell, and DC	2023^103^
HADH	KIRC	B cell, M2 macrophages, and DC	Helper T cell, Tregs, and neutrophil	2021^104^

ACLY, adenosine triphosphate–citrate lyase; ACSS2, acetate by acetyl-coenzyme A synthetase 2; HADH, L-3-hydroxyacyl-coenzyme A dehydrogenase; ACACA, acetyl-coenzyme A carboxylase alpha; HCC, hepatocellular carcinoma; CESC, cervical squamous cell carcinoma and endocervical adenocarcinoma; KIRC, kidney renal clear cell carcinoma; CD, cluster of differentiation; Th, T-helper; DC, dendritic cell; NK, natural killer; Tregs, T-regulatory; Tcm, T-central memory; mDC, myeloid dendritic cell; TME, tumor microenvironment.

In recent years, many *in vivo* studies utilizing different animal models have been conducted to validate the analysis results of key FA-associated metabolic targets or explore the effects of FA-associated genes on immune cell composition with targeted drugs or gene-editing technology (Table 3). For instance, decreased uptake of FAs by inhibiting CD36 and FABP1 in tumor models presented better survival with positive anti-tumoral immune changes.^105^^,^^106^ Targeting FA *de novo* synthesis or oxidation enzymes could also inhibit tumor growth.^80^^,^^107^, ^108^, ^109^, ^110^

**Table 3:** Key targets in FA metabolism and the TME changes *in vivo*.

Target	Tumor Model	Treatment	Tumor control	TME changes	Year
CD36	Lung carcinoma, colon cancer	KO	Better	Decreased CD8^+^T cells and MDSCs	2017^105^
FABP1	Hepatocellular carcinoma	KO; inhibitor + anti-PD-1	Better	Increased DCs, M1 macrophages and B cells; decreased Tregs and NK cells	2023^106^
FABP5	Lung metastasis	KO	Worse	Lower activation of NK cells	2021^111^
ACLY	Breast cancer, lung cancer	Inhibitors	Better	Treg depletion	2016^107^
ACSS2	Breast cancer	KO; inhibitor	Better	Increased CD4^+^ T cells, CD8^+^ T cells, and NK cells	2023^80^
ACC	Fibrosarcomas, melanomas, adenocarcinomas	Inhibitor	Better	Increased memory trait and decreased exhaustion in T cell	2024^108^
FASN	Breast cancer	Inhibitor + PI3Kα inhibitor	Better	Increased CD8^+^ T and CD4^+^T cells; decreased M2 macrophages	2021^112^
	Melanoma metastasis	Inhibitor	Better	Increased DCs, NK cells and CD8^+^T cells; decreased Tregs	2020^109^
ACSL3	Pancreatic ductal adenocarcinoma	KO	Better	Decreased fibroblasts, M2 macrophages and Tregs; increased CD8^+^T cells	2020^110^
ACSL4	Melanoma	KO + PD1 antibody	Better	Increased CD8^+^ T and CD4+T cells	2022^113^
CPT1A	Lung cancer	KO + PD1 antibody	Better	Increased CD8^+^ T cells and decreased M2 macrophages	2024^114^
	Breast cancer	KO + ErbB2 antibody	Better	Increased CD8^+^ T cells, NK cells and M1 macrophage	2024^115^

CD, the cluster of differentiation; FABP, FA-binding protein; ACLY, adenosine triphosphate–citrate lyase; ACSS2, acetate by acetyl-coenzyme A synthetase 2; ACC, acetyl-coenzyme A carboxylase; FASN, FA synthase; ACSL, long-chain acyl-coenzyme A synthetase; CPT, carnitine acyl transferase; TME, tumor microenvironment; MDSC, myeloid-derived suppressor cell; DC, dendritic cell; NK, natural killer; Tregs, T-regulatory; PD-1, programmed cell death protein 1; ErbB2, erb-b2 receptor tyrosine kinase 2; PI3K, phosphoinositide 3-kinase.

### FA metabolism in innate immune cells in TME

#### Macrophages

In the TME, macrophages typically present two distinct phenotypes, namely M1-like TAMs (pro-inflammatory and anti-tumoral) and M2-like TAMs (anti-inflammatory and pro-tumoral).^116^ Both phenotypes exhibit differences in their functions, secreted cytokines, surface markers, and metabolic requirements, and they act contrarily in tumor progression.^117^ Up-regulated aerobic glycolysis and increased glutamine decomposition contribute to lipogenesis in M1-like TAMs.^118^ In contrast, M2-like TAMs depend mainly on up-regulated FA oxidative phosphorylation (OXPHOS) and β-oxidation to generate ATP, which is a consequence of FA accumulation in the TME.^1^

Compared with general macrophages, TAMs exhibit increased lipid uptake owing to upregulated CD36 expression.^119^ Zhang et al. showed up-regulated E-FABP expression in MHCII^+^CD11c^+^ TAMs, which presented an M1-like phenotype.^120^ A-FABP has been reported to be up-regulated in macrophages cocultured with breast cancer cells, along with interleukin (IL)-6/signal transducer and activator of transcription (STAT)3 signaling activation.^121^ Reportedly, peroxisome proliferator-activated receptor (PPAR)γ promotes M2 polarization of TAMs.^122^, ^123^, ^124^ In an IL-4-induced macrophage M2 polarization process, it was shown that the IL-4/S100A4/PPARγ/CD36 axis promoted FAO in macrophages.^125^

**Figure 2:**
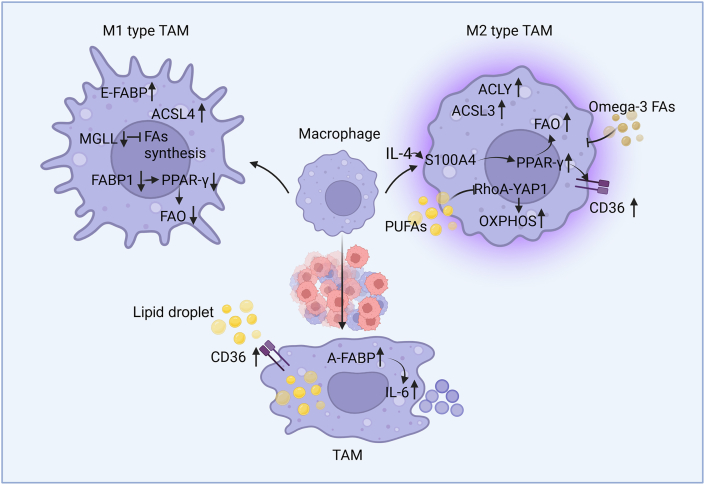
FA metabolic alterations trigger the switch of the M2-to-M1 phenotype in TAMs. Macrophages exhibit decreased MGLL and FABP1 expression to inhibit FA synthesis and FAO during M1 type polarization. Omega-3 FAs inhibit M2-like TAM infiltration while polyunsaturated FAs (PUFAs) promote M2-like TAM polarization by increasing OXPHOS. M1 type TAMs display increased E-FABP and ACSL4 expression, while M2-like TAMs exhibit increased ACLY and ACSL3 expression. When co-cultured with cancer cells, macrophages up-regulate the CD36 and A-FABP expression. FA, fatty acid; TAM, tumor-associated macrophage; FABP, FA-binding protein; E-FABP, epidermal FABP; ACSL, long-chain acyl-coenzyme A synthetase; MGLL, monoglyceride lipase; PPAR, peroxisome proliferator-activated receptor; FAO, FA oxidation; CD, cluster of differentiation; A-FABP, adipocyte FABP; IL, interleukin; ACLY, adenosine triphosphate–citrate lyase; YAP1, yes-associated protein 1; OXPHOS, oxidative phosphorylation.

Notably, targeting FA metabolism, especially OXPHOS and FAO in macrophages, can trigger the switch of the M2-to-M1 phenotype in the TME (Fig. 2). For instance, omega-3 FAs have been shown to inhibit M2-like TAM infiltration and delay orthotopic tumor growth.^126^ In ovarian cancer, polyunsaturated FAs could promote M2-like TAM polarization, resulting in increased OXPHOS owing to the down-regulation of RhoA-yes-associated protein 1 signaling.^127^ In HCC, FABP1 deficiency in TAMs could revert the pro-tumor M2 phenotype to M1^106^ and possibly restrict HCC development by increasing TAM FAO via the FABP1–PPARγ interaction. In a study, the pseudogene transmembrane protein 198B/pleomorphic adenoma gene-like 2/ACLY pathway activated ACLY, and the indirect increase of ACLY induced M2 polarization of macrophages.^128^ In contrast, CD40 activation in macrophages promoted the M1 phenotype by increasing ACLY-dependent histone acetylation of pro-inflammatory genes.^129^ In another study, cathepsin inhibitor-treated M2/M0 macrophages exhibited an increased expression of FASN and pro-inflammatory mediators.^130^ ACSL3 deletion in pancreatic cancer mice model could decrease the number of M2-polarized macrophages,^110^ whereas ACSL4 overexpression simultaneously increased M1 polarization and reduced M2 polarization in macrophages via ferroptosis.^131^ Metabolic supramolecular nano-particles (MSNPs) loaded with FAO inhibitors and Toll-like receptor (TLR)7/8 agonists reprogrammed macrophages from the M2 phenotype to M1^132^. Cao et al. employed a nanoplatform-based co-delivery of monoglyceride lipase and cannabinoid receptor 2 small-interfering RNA (siRNA) systems to suppress free FA generation and thus repolarized TAMs into M1-like phenotype.^133^

#### Myeloid-derived suppressor cells

Myeloid-derived suppressor cells (MDSCs) are a heterogeneous population of immature myeloid cells that can be subdivided into monocytic (M)-MDSCs and polymorphonuclear (PMN)-MDSCs based on their morphology and phenotype. These cells can suppress the anti-tumor immune response to facilitate tumor progression via various mechanisms.

Tumor-infiltrating MDSCs up-regulate FAO and the expression of related genes, including, CPT1, medium-chain acyl-CoA dehydrogenase, PPAR gamma coactivator 1β, and HADHA. The combined effect of FAO inhibition and low-dose chemotherapy has been shown to effectively down-regulate the immunosuppressive effects of MDSCs in tumor models.^134^

FATP2 is particularly overexpressed in PMN-MDSC, with a higher expression resulting in increased lipid accumulation and decreased activation of PMN-MDSC via granulocyte-macrophage colony-stimulating factor/phosphorylated STAT5.^135^ Additionally, the proviral integration site for the Moloney murine leukemia virus 1/PPARγ axis has been reported to regulate FAO metabolism, CD36 expression, and immunosuppressive function in MDSCs.^136^ Free FA receptor 2 (FFAR2) is highly expressed in MDSCs and is associated with MDSC accumulation in lung tumors. FFAR2 deficiency in M-MDSCs can impair their immunosuppressive activity through the Gαq/calcium/PPAR-γ axis.^137^

#### Dendritic cells

Dendritic cells (DCs) are specialized antigen-presenting cells (APCs) involved in the activation and maintenance of anti-tumor immunity.^138^ They are mainly divided into three groups: classical DCs (cDCs) type 1 (cDC1) and type 2, and plasmacytoid DCs (pDCs). cDCs activate T cells and contribute greatly to the TME, especially cDC1s, which present antigens to CD8^+^ T lymphocytes.^139^

In tumor-bearing mice and patients with cancer, DCs have been reported to show higher lipid accumulation than that in healthy individuals via up-regulated FA uptake, and DCs with high lipid levels could not present tumor-associated antigens to activate T cells.^138^ In CRC models, loss of FFAR2 induced overactivation, increased DC death, and increased exhaustion of CD8^+^ T cells.^140^ The accumulation of saturated FAs (such as PAs) rather than unsaturated FAs (such as oleic acid) in APCs has been reported to inhibit APC–T-cell conjugation and reduce major histocompatibility complex (MHC-I) surface expression.^141^ Additionally, ovarian cancer-associated DCs have demonstrated higher levels of intracellular lipids and reactive oxygen species than control DCs do.^142^ In HCC, DCs cocultured with α-fetoprotein exhibited reduced FA synthesis and mitochondrial OXPHOS.^143^

#### Natural killer cells

Natural killer (NK) cells are a group of innate lymphocytes that can exhibit cytotoxicity and produce cytokines in response to malignant cells. Upon activation by lymphoma, NK cells upregulate FAO and CPT1A expression, which is essential to their mitochondrial function.^144^ CPT1A then promotes the activity of NK cells against cancer cells and has been shown to protect their function both *in vivo* and *in vitro*.^144^^,^^145^ In aggressive B-cell lymphoma, increased PPAR-γ expression has been observed in NK cells. However, excessive exposure to FAs has been reported to suppress NK cell metabolism and function, which is contrary to the findings of other studies and is attributed to be a functional adaptation to the lymphoma environment.^146^

### FA metabolism in adaptive immune cells in the TME

#### T cells

The behavior of different T cell subtypes varies in response to lipid overaccumulation. Various lipid metabolism-associated genes facilitate the utilization of essential nutrients in cytotoxic T lymphocytes (CTLs), thereby promoting anti-tumor immunity, whereas they can facilitate immune evasion of T-memory (T_mem_) and T-rgulatory cells (Tregs).^147^

**Figure 3:**
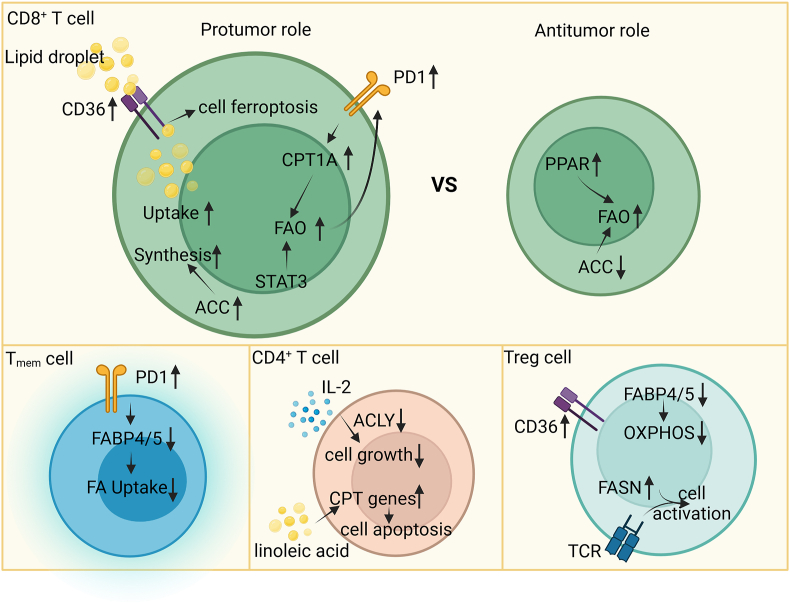
FA metabolic changes of T cells in the TME. Elevated levels of CD36, ACCs and CPT1A facilitate FA uptake, synthesis and oxidation in CD8^+^ T cells to promote tumor progression. FA metabolic gene and protein regulation in T_mem_ cells and CD4^+^ T cells, together with FASN-mediated Treg cell activation contributes to the shaping of immunosuppression in the TME. CD, cluster of differentiation; PD1, programmed cell death protein 1; CPT, carnitine acyl transferase; FAO, FA oxidation; STAT, signal transducer and activator of transcription; ACC, acetyl-coenzyme A carboxylase; PPAR, peroxisome proliferator-activated receptor; FABP, FA-binding protein; T_mem_, T-memory; IL, interleukin; ACLY, adenosine triphosphate–citrate lyase; Treg, T-regulatory; OXPHOS, oxidative phosphorylation; FASN, FA synthase; TCR, T-cell receptor.

Increasing evidence suggests the pro-tumor role of FA reprogramming in CD8^+^ TILs, characterized by increased FA uptake, synthesis, and oxidation (Fig. 3). In both multiple myeloma and pancreatic ductal adenocarcinoma, excessive LCFA uptake has been shown to suppress CD8^+^ T-cell function.^148^^,^^149^ The uptake of oxidized lipids owing to increased CD36 expression promotes CD8^+^ T cell lipid peroxidation and dysfunction in tumors.^150^ Reportedly, FAO in CD8^+^ T cells is considerably increased in the TME, suggesting a complex role of T cells in the metabolic regulation of cancer.^151^ Programmed cell death protein 1 (PD-1) promotes FAO in CD8^+^ T cells by increasing CPT1A expression and STAT3 activation, which has been shown to promote breast tumorigenesis.^152^^,^^153^ In a study, blocking lipid oxidation with ranolazine promoted CD8^+^ T-cell cytotoxic activity and decreased PD-1 expression.^154^ Another study showed that inhibiting ACC promoted cell survival, as it allowed CD8^+^ T cell to utilize lipids.^108^ Although these studies indicate that FAO interferes with the anti-tumor function of CD8^+^ T cells, other studies have reported that PPAR-induced FAO can promote T-cell functions and effectively function with PD-1 blockade in a collaborative manner.^151^^,^^155^ In mechanism, CD8^+^ T-cell dysfunction can be caused by CD36-mediated ferroptosis.^156^

Notably, the infiltrating tissue-resident T_mem_ cells rely on FAO for survival, and programmed death-ligand 1 (PD-L1) inhibitors have been shown to increase FABP4/5 expression to promote lipid uptake in T_mem_ cells, enhancing its survival in gastric adenocarcinoma.^157^ Additionally, CD36 deficiency can impair the accumulation and immunosuppressive function of intra-tumoral Tregs and stimulate their apoptosis.^158^ Reportedly, FABP5 inhibition induced mitochondrial changes such as loss of cristae structure due to decreased OXPHOS and impaired lipid metabolism, thereby promoting the suppressive activity of Tregs.^159^ Lim et al. showed that FASN loss reduced the function of T-cell receptor-dependent active Tregs and inhibited tumor growth.^160^ In CD4^+^ T lymphocytes, ACLY could act as a critical phosphoprotein during IL-2-promoted T-cell growth in anti-cancer immunotherapies.^161^ Linoleic acid-induced CPT gene expression in HCC has been shown to promote CD4^+^ T cell apoptosis and tumor formation.^162^

#### B cells

Owing to a low composition of B cells in solid tumors, most FA metabolism-related studies on B cells are focused on blood cancers. As reported in colorectal cancer, accelerating FAO in B cells inhibited their switch to the IgA class, which prevented cancer progression.^163^ In aggressive B-cell lymphoma, indirect inhibition of FATP1 or CPT1A significantly reduced cell viability and proliferation.^164^ CD36 and FASN overexpression was found in diffuse large B-cell lymphoma (DLBCL) and was correlated with drug resistance.^165^ Besides, inhibiting FASN and HADHA negatively regulated DLBCL cell growth,^166^^,^^167^ but the activation of ACSL4 promoted cell ferroptosis in the GCB subtype of DLBCL.^168^ In chronic lymphocytic leukemia (CLL), B-lymphocytes showed a high level of FAO via CPT1A, which was a vital cause for cancer progression and drug resistance.^169^^,^^170^

### FA metabolism in non-immune cells in TME

#### Cancer-associated fibroblasts

Cancer-associated fibroblasts (CAFs) are active fibroblasts that secrete cytokines, matrix proteins, and immunomodulatory factors to support cancer cell growth and metastatic dissemination.^171^ Compared with normal fibroblasts, CAFs reportedly underwent lipidomic reprogramming and excreted more FA, enhancing CRC cell migration.^172^ Similarly, compared with breast fibroblasts, CAFs exhibited a higher PDK1 expression under normoxia and lower expression under hypoxic conditions.^80^

Compared with young fibroblasts, when cocultured with aged fibroblasts, melanoma cells showed up-regulated FATP2 expression and increased lipid uptake.^76^ CD36 inhibition in CAFs has been shown to negatively affect their proliferation and migration abilities.^173^ Pancreatic CAFs have been shown to secrete ACLY-dependent acetate to promote cancer development.^174^ In OSCC, phosphorylated ACLY (p-ACLY) was up-regulated in CAFs, and lipid synthesis and accumulation were up-regulated.^175^ Furthermore, CAFs have been shown to promote the proliferation, migration, and invasion of colon cancer cells via CPT1A up-regulation to increase FAO.^176^ In a study, the inhibition of FA metabolism by FASN siRNA or CD36 antibody resulted in the inhibition of CAF-induced CRC cell migration.^172^

#### Adipocytes

Adipocytes are a part of various TMEs and can promote tumor progression via the communication between adipocytes and tumor cells, especially lipid exosomes.^177^, ^178^, ^179^ For instance, melanoma cells exhibited enhanced growth and invasion when adipocyte-derived lipids were transferred to cancer cells via FATPs.^180^ Adipocyte exosomes carrying FAO-associated proteins increased FAO in melanoma both *in vitro* and *in vivo*.^181^ Furthermore, breast cancer cells co-cultivated with adipocytes showed increased lipolysis and adipose triglyceride lipase, and FABP5 up-regulation, resulting in enhanced migration and invasion abilities.^182^

#### Endothelial cells

Activated endothelial cells (ECs) primarily rely on glycolysis rather than glucose oxidation and FAO to produce ATP, although FAO is required for DNA replication and proliferation during vascular sprouting in ECs.^183^ Reportedly, neurogenic locus notch homolog protein 1-mediated FABP4 downregulation in ECs in ovarian cancer increased FAO and decreased angiogenesis.^184^ In contrast, IL-17A-stimulated endothelial FAO increased mitochondrial respiration and angiogenesis.^185^ Notably, FASN inhibitors have been reported to impair angiogenesis via mammalian target of rapamycin malonylation.^186^

#### Astrocytes

In astrocytes, FABP7 has been shown to interact with ACLY in the nucleus to regulate caveolin-1 transcription and acetyl-CoA metabolism.^187^

### FA metabolism in TME crosstalk

#### Crosstalk between tumor and non-tumor cells

In the TME, different tumor and non-tumor cells are in constant crosstalk with each other, exhibiting intricate cellular communication. For instance, in the crosstalk with tumor cells, macrophages up-regulated CD36 expression to increase tumor cell-mediated FA uptake and lipid deposition, along with tumor-derived lipid-driven macrophage M2 polarization in liver metastasis.^188^ In bladder cancer, tumor-derived exosomal circRNA_0013936 inhibited the functions of CD8^+^ T cells by promoting FATP2 expression in PMN-MDSCs.^189^ DCs derived from FASN^high^ ovarian tumor-bearing mice presented higher levels of lipid and lipid accumulation, which impaired their antigen presentation activity and T cell activation.^190^ In lung cancer, TAM-derived l-carnitine induced CPT1A-mediated cancer cell ferroptosis resistance.^114^ Wnt5a derived from melanoma up-regulated DC FAO via the PPAR-γ–CPT1A axis to promote Treg differentiation.^191^ Similarly, FA-carrying tumor-derived exosomes mediated DC dysfunction by increasing FA uptake and lipid accumulation via the PPARα-mediated FAO mechanism.^192^

**Figure 4:**
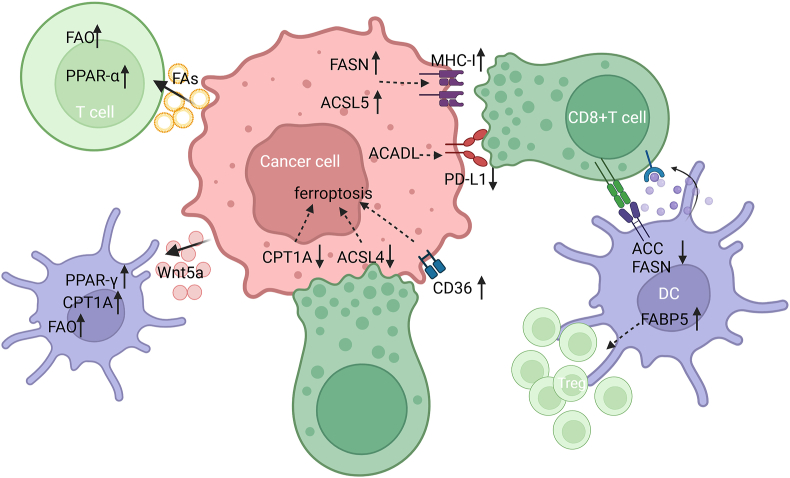
FA-related cell interactions among cancer cells, T cells, and dendritic cells. Cancer cell-derived FAs and Wnt5a respectively induce elevated FAO levels in T cells and dendritic cells. Increased MHC-I and decreased PD-L1 expression in cancer cells, which is mediated by FASN, ACSL5 and ACADL, promote CD8^+^ T cell cytotoxicity. The expression of CPT1A, ACSL4 and CD36 in cancer cells also regulate CD8^+^ T-cell-induced tumor ferroptosis. ACC, FASN and FABP5 in DCs exert an impact on their interaction with CD8^+^ T cells and Treg cells. FA, fatty acid; FAO, FA oxidation; CD, cluster of differentiation; PPAR, peroxisome proliferator-activated receptor; CPT, carnitine acyl transferase; FASN, FA synthase; ACSL, long-chain acyl-coenzyme A synthetase; ACADL, long-chain acyl-coenzyme A dehydrogenase; MHC, major histocompatibility complex; PD-L1, programmed death-ligand 1; ACC, acetyl-coenzyme A carboxylase; DC, dendritic cell; FABP, FA-binding protein; Treg, T-regulatory.

Notably, FA metabolic reprogramming changes surface molecule expression on cancer cells to increase their susceptibility to immune checkpoint blockade therapy (Fig. 4). For instance, FASN inhibition in HCC increased MHC-I expression and promoted the anti-tumor activity of CD8^+^ T cells. Furthermore, FASN inhibitors and anti-PD-L1 antibodies could combinedly suppress HCC tumor growth *in vivo*.^193^ ACSL5 expression has been shown to up-regulate MHC-I antigen expression on the tumor cell surface to being sensitized to CD8^+^ T-cell-mediated cytotoxicity.^194^ In a study, CPT1A loss sensitized melanoma cells to CD8^+^ T-cell killing and rendered metastatic melanoma more susceptible to T-cell therapy.^195^ In another study, ACADL could suppress PD-L1 expression in LUAD cells and aggravated T cell-induced cytolysis.^56^ Many studies have reported the relationship between FA metabolism and CD8^+^T-cell-mediated cancer cell ferroptosis. Notably, owing to the high cystine demands of cancer cells, cystine deprivation can induce CD36-mediated FA accumulation and ferroptosis in CD8^+^ T cells.^196^ Additionally, CPT1A acts as an essential driver in CD8^+^ T-cell-mediated resistance to ferroptosis in lung cancer stem cells.^114^ Similarly, T-cell-derived interferon (IFN)-γ, in combination with arachidonic acid, has been shown to promote tumor ferroptosis in an ACSL4-dependent manner.^197^ Saturated FAs have been shown to inhibit the stimulator of the interferon genes–IFN–I pathway in cancer cells and induce T cell activation in head and neck squamous cell carcinomas.^198^

**Figure 5:**
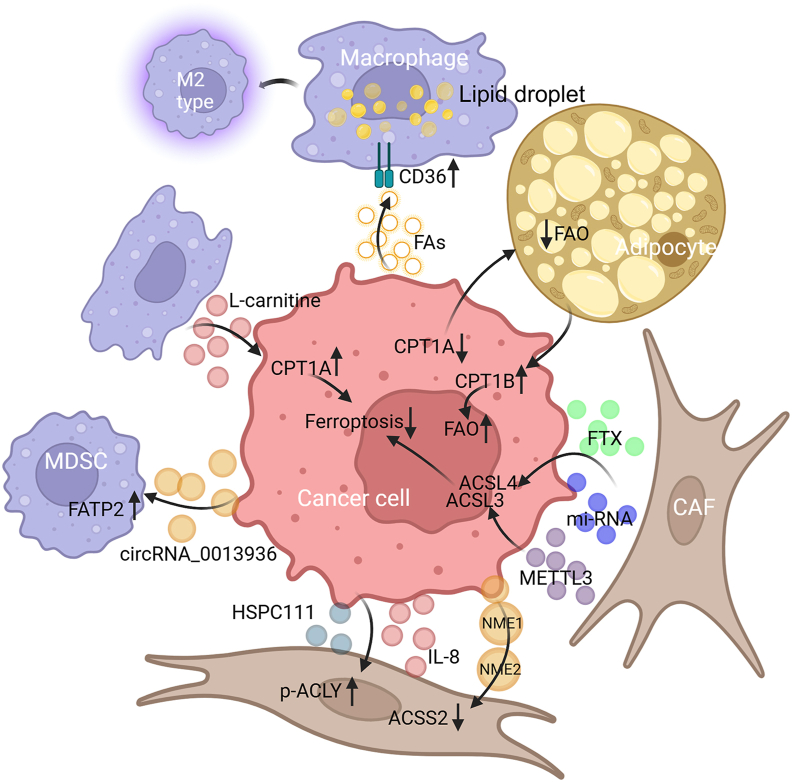
FA-related cell interaction between cancer and non-cancer cells. Cancer cell derived extracellular vesicles containing FAs, circRNA_0013936 and several proteins (HSPC111, IL-8 and NME1/2) alter FA-related enzyme expression in macrophages, MDSCs and CAFs. Macrophage secreted l-carnitine and CAFs-derived exosomes change cancer cell FA metabolism to inhibit ferroptosis. Down-regulation of CPT1A in cancer cells decreases adipocyte FAO, but cancer cells exhibit increased FAO via increased CPT1B when co-cultured with adipocytes. FA, fatty acid; FAO, FA oxidation; MDSC, myeloid-derived suppressor cell; FATP, FA transport protein; CD, cluster of differentiation; p-ACLY, phosphorylated adenosine triphosphate–citrate lyase; ACSS2, acetyl-coenzyme A synthetase 2; IL, interleukin; CPT, carnitine acyl transferase; ACSL, long-chain acyl-coenzyme A synthetase; CAF, cancer-associated fibroblast; METTL3, methyltransferase 3, N6-adenosine-methyltransferase complex catalytic subunit; NME, nucleoside diphosphate kinase.

Exosomal interactions between cancer cells and CAFs have been reported to promote tumor progression (Fig. 5). For instance, CRC-derived exosomal HSPC111 could increase ACLY phosphorylation in CAFs, thereby promoting tumor liver metastasis.^199^ OSCC cells-derived IL-8 enhanced lipid synthesis in CAFs via the protein kinase B/p-ACLY axis.^175^ ACSS2 expression was shown to be suppressed when human fibroblasts were treated with NME1/2-carrying breast carcinoma cell-derived extracellular vesicles.^200^ In contrast, METTL3 in CAF-derived exosomes promoted CRC cell proliferation and metastasis by suppressing ACSL3-m6A-modification-induced ferroptosis.^201^ Similarly, CAF exosome-derived microRNA could target ACSL4 and inhibit ferroptosis in pancreatic cancer cells.^202^ Podoplanin^+^ CAFs could also inhibit cell ferroptosis and promote OSCC invasiveness by down-regulating ACSL4 expression.^203^

In colon cancer cells, CPT1A knockdown inhibited the tumor-promoting effect of adipocytes by attenuating FA utilization.^204^ Similarly, gastric cancer cells showed enhanced FA oxidation after coculture with adipocyte, which was mediated by CPT1B.^205^

#### Crosstalk among non-tumor cells

In the TME, various non-tumor cells also present an intricate crosstalk among them. For instance, high expression of E-FABP in TAMs has been shown to promote lipid droplet generation and IFN-β secretion, promoting the recruitment of anti-tumoral cells, especially NK cells.^120^ Furthermore, lipid droplets in macrophages can effectively prolong lipid droplet-laden macrophage survival and enhance chemokine ligand 20 secretion to recruit Tregs in HCC.^206^ In a study, Tregs promoted sterol regulatory element binding protein 1-mediated FA synthesis in M2-like TAMs by suppressing CD8^+^ T cell-derived IFN-γ secretion.^207^ Similarly, FFAR2 inhibition in BM-MDSCs also reduced CD8^+^ T cell recruitment and cytotoxicity and enhanced anti-PD-1 therapy.^137^

Herber et al. reported that an ACC inhibitor improved the ability of DCs to stimulate allogeneic T cells.^138^ FABP5 expression in tumor-associated pDCs further supported the generation of Tregs.^208^ In tumor-associated DCs, X-box binding protein 1 activation induced abnormal lipid accumulation and inhibited the capacity of DCs to support anti-tumor T cells.^142^

**Figure 6:**
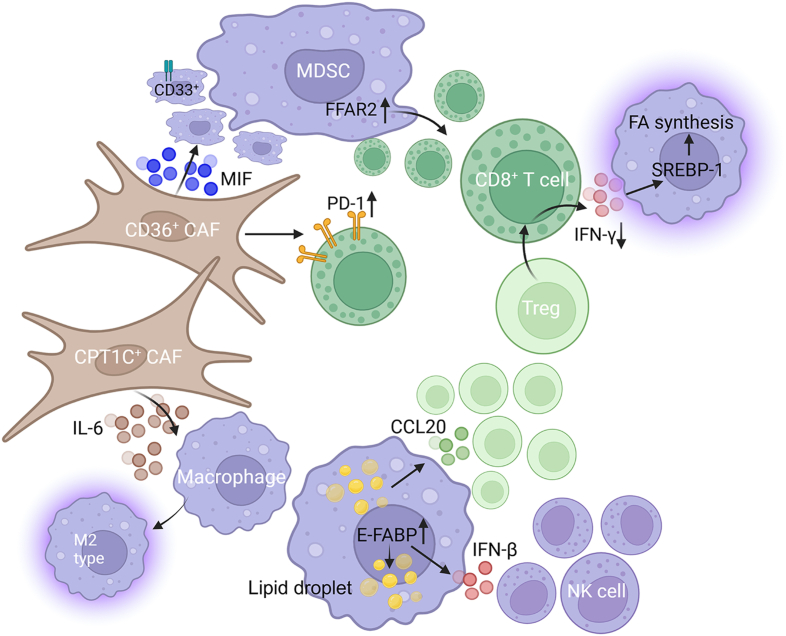
FA-related cell interactions among non-cancer cells. CD36^+^ CAFs affect CD33^+^ MDSC recruitment and PD-1 expression in CD8^+^ T cells. CPT1C^+^ CAFs promote macrophage M2-like polarization by secreting IL-6. Lipid droplets in macrophages promote Treg cell and NK cell recruitment via elevated CCL20 and IFN-β secretion, respectively. FFAR2 expression in MDSCs has an influence on CD8^+^ T cell recruitment and cytotoxicity, meanwhile, CD8^+^ T cell-derived IFN-γ regulates SREBP-1 expression in M2-like TAMs. FA, fatty acid; MDSC, myeloid-derived suppressor cell; CD, cluster of differentiation; MIF, migration inhibitory factor; FFAR2, free fatty acid receptor 2; SREBP-1, sterol regulatory element binding protein 1; CAF, cancer-associated fibroblast; CPT1C, carnitine palmitoyltransferase 1C; IL, interleukin; CCL20, chemokine ligand 20; E-FABP, epidermal FA-binding protein; IFN, interferon, NK, natural killer; PD-1, programmed cell death protein 1.

Additionally, CPT1C^+^ CAFs have been shown to promote M2-like phenotype of macrophages by up-regulating IL-6 in gastric cancer.^209^ CD36^+^ CAFs could recruit CD33^+^ MDSCs via secretion of macrophage migration inhibitory factor to provide an immunosuppressive microenvironment in HCC.^210^ Similarly, CD36^+^ CAFs affected PD-1 expression in CTLs to promote CTL exhaustion^173^ (Fig. 6).

## Therapeutic implications

Lipid metabolism reprogramming, especially FA metabolism, is becoming an increasingly potential mechanism in the development of anti-tumor therapies. Therefore, we summarized recent therapies targeting FA metabolism in preclinical and clinical research to identify potential drugs for cancer treatment (Table 4). Only a few FA-metabolism inhibitors have been applied in clinical trials, including VT1021(CD36 inhibitor), MTB-9655 (ACSS2 inhibitor), and TVB-2640(FASN inhibitor). Meanwhile, some other inhibitors, such as SSO, SB-204990, TVB-3664, and Etomoxir, have been used in many studies on various tumor-bearing animal models and have shown strong anti-tumor effect, which can be selected as candidates for subsequent clinical trials.

**Table 4:** Therapies targeting FA metabolism in preclinical and clinical research.

Target	Drug	Phase	Tumor type	Reference
CD36	SSO	Preclinical	Breast, Ovarian, Colorectal, Cervical cancer	27,28,212,213
	2-Methylthio-1,4-naphthoquinone (MTN, small molecule)	Preclinical	Glioblastoma	26
	FA6.152	Preclinical	Breast cancer, OSCC	27,32
	JC63.1	Preclinical	OSCC, Breast, Ovarian, Gastric cancer	27,30,32,214
	Cyclic psap	Preclinical	Ovarian cancer	215
	VT1021	Phase 2–3 (NCT03364400)	Glioblastoma, Breast, Ovarian, Pancreatic cancer	216,217
		(NCT03970447)		
FABPs	SBFI-102/103/26 (FABP5 inhibitor)	Preclinical	Prostate cancer	72,73
	BMS309403 (FABP4 inhibitor)	Preclinical	Ovarian cancer, Cholangiocarcinoma	74,75
FATPs	Lipofermata (FATP2 inhibitor)	Preclinical	Melanoma	76,180
ACLY	SB-204990	Preclinical	Melanoma, Lung, Ovarian cancer	77,218,219
	Bempedoic acid	Preclinical	HNSCC, Colorectal, Pancreatic cancer	78,220,221
	BMS-303141	Preclinical	ESCC	79,222
ACSS2	VY-3-135	Preclinical	Breast cancer	80
	ACSS2 inhibitor	Preclinical	Prostate cancer	223
	A small-molecule inhibitor	Preclinical	Breast cancer	224
	AD-8007 5584	Preclinical	Breast cancer	81
	1-(2,3-di(thiophen-2-yl)quinoxalin-6-yl)-3-(2-methoxyethyl) urea	Preclinical	Bladder cancer	222
	MTB-9655	Phase 1 (NCT04990739)	Advanced solid tumor	82
ACC	TOFA	Preclinical	Breast cancer	83
	PF-05175157	Preclinical	HCC	84
FASN	TVB-2640	Phase 1–3 (NCT03032484)	Astrocytoma, Glioblastoma, NSCLC, Breast, Ovarian cancer	85,225
		(NCT05118776)		
		(NCT03179904)		
		(NCT02223247)		
	TVB-3166	Preclinical	Gastric, Bladder cancer	86,226
	TVB-3664	Preclinical	Melanoma, HCC, Pancreatic, Lung, Colorectal cancer	87,88,227, 228, 229
	Orlistat	Preclinical	HCC, Colorectal, Pancreatic cancer	88,193,230, 231, 232, 233, 234
	Cerulenin	Preclinical	Liver, Cervical cancer	89,235
	C75	Preclinical	Breast, Cervical, Glioblastoma, Gastrointestinal stromal cancer	89,112,236, 237, 238
ACSLs	Triacsin C	Preclinical	Glioblastoma, Colorectal cancer	92,239
CPT1	Etomoxir	Preclinical	Glioblastoma, Melanoma, HCC, Breast, Colorectal, Colon, Ovarian cancer	90,176,240, 241, 242, 243, 244, 245, 246
	Perhexiline	Preclinical	Glioblastoma, Pancreatic, Ovarian cancer	91,247,248

OSCC, oral squamous cell carcinoma; HNSCC, head and neck squamous cell carcinoma; ESCC, oesophageal squamous cell carcinoma; HCC, hepatocellular carcinoma; CD, the cluster of differentiation; FABP, FA-binding protein; ACLY, adenosine triphosphate–citrate lyase; ACSS2, acetate by acetyl-coenzyme A synthetase 2; ACC, acetyl-coenzyme A carboxylase; FASN, FA synthase; ACSL, long-chain acyl-coenzyme A synthetase; CPT, carnitine acyl transferase.

Although clinical research is lacking, various *in vivo* and *in vitro* studies have shown increased efficacy of immunotherapy, especially T cell therapy, in combination with FA enzyme inhibitors. A review on lipid metabolic reprogramming in the TME has delivered a detailed summary of the attempts to combine FA-targeted (such as CD36, FATP2, FASN, CPT, and PPAR) therapies with immunotherapy published before 2023.^211^ Considered as a research hotspot, many studies on this kind of combination therapy, which will be supplemented as follows, are still being developed in recent years. Pharmacologic inhibition of CPT1A sensitized melanoma cells to chimeric antigen receptor-T therapy.^195^ Similarly, FABP1, ACSL4, CPT1A, FASN, and FFAR2 inhibitors can also enhance anti-PD-1 therapies in cancers.^106^^,^^113^^,^^114^^,^^137^^,^^193^ Additionally, the use of delivery systems such as MSNPs loaded with a TLR7/8 agonist and FAO inhibitor to reduce tumor progression *in vivo* via macrophage phenotype reprogramming^132^ is an approach with significant therapeutic potential. Consequently, exploring and identifying more targets for such combinatorial strategies and validating their potential in clinical trials is crucial in future studies.

## Conclusions and perspectives

FAs play a vital role in tumor progression, not only in the intracellular process of cancer cells but also in cellular interactions via soluble proteins and exosomes. Furthermore, FA metabolic reprogramming in non-cancer cells contributes to the formation and maintenance of immunosuppressive TME.

Clarifying FA metabolic changes in non-cancer cells can help to enhance the efficacy of metabolic-targeted treatment and immunotherapy. However, the cell metabolic pattern of immune cells varies from different immune cells or cell subtypes, which makes it a problem to find an accurate target for all cell compositions. Besides, due to the general existence of FA metabolic pathway in normal tissues, nonspecifically inhibiting FA-related enzymes may cause severe toxicity on certain organs, such as muscle and liver. Consequently, we should develop more tumor-specific inhibitors and optimize the drug delivery systems to improve the accuracy, efficacy, and reduce side effects of metabolic cancer therapies.

Considering the intricate interplay of TME components, future studies should focus on elucidating the functions and mechanisms of cell co-adaptation in response to nutrient depletion. Advances in these research areas may provide insights regarding immunotherapy resistance and the development of novel combinatorial strategies with FA metabolism inhibitors.

## CRediT authorship contribution statement

**Wenxin Zhang:** Writing – original draft, Investigation, Writing – review & editing, Methodology, Visualization, Conceptualization. **Peiwen Wang:** Visualization, Methodology, Writing – original draft, Resources, Software, Investigation. **Guiqiang Yuan:** Resources, Software, Methodology. **Fusheng Liu:** Supervision. **Guishan Jin:** Supervision, Writing – review & editing. **Junwen Zhang:** Writing – review & editing, Conceptualization.

## Funding

This work was supported by the Beijing Natural Science Foundation (China) (No. 7222019), and the Beijing Laboratory of Biomedical Materials Foundation (China).

## Conflict of interests

The authors declare no competing interests.
